# Identification and Expression Analysis of WRKY Gene Family in Response to Abiotic Stress in *Dendrobium catenatum*

**DOI:** 10.3389/fgene.2022.800019

**Published:** 2022-02-03

**Authors:** Tingting Zhang, Ying Xu, Yadan Ding, Wengang Yu, Jian Wang, Hanggui Lai, Yang Zhou

**Affiliations:** ^1^ Key Laboratory for Quality Regulation of Tropical Horticultural Crops of Hainan Province, School of Horticulture, Hainan University, Haikou, China; ^2^ Hainan Key Laboratory for Biotechnology of Salt Tolerant Crops, School of Tropical Crops, Hainan University, Haikou, China

**Keywords:** *Dendrobium catenatum*, WRKY transcription factors, gene family, gene expression, abiotic stress

## Abstract

*Dendrobium catenatum* has become a rare and endangered medicinal plant due to habitat loss in China. As one of the most important and largest transcription factors, WRKY plays a critical role in response to abiotic stresses in plants. However, little is known regarding the functions of the WRKY family in *D. catenatum*. In this study, a total of 62 *WRKY* genes were identified from the *D. catenatum* genome. Phylogenetic analysis revealed that DcWRKY proteins could be divided into three groups, a division supported by the conserved motif compositions and intron/exon structures. *DcWRKY* gene expression and specific responses under drought, heat, cold and salt stresses were analyzed through RNA-seq data and RT-qPCR assay. The results showed that these genes had tissue-specificity and displayed different expression patterns in response to abiotic stresses. The expression levels of *DcWRKY22*, *DcWRKY36* and *DcWRKY45* were up-regulated by drought stress. Meanwhile, *DcWRKY22* was highly induced by heat in roots, and *DcWRKY45* was significantly induced by cold stress in leaves. Furthermore, *DcWRKY27* in roots and *DcWRKY58* in leaves were extremely induced under salt treatment. Finally, we found that all the five genes may function in ABA- and SA-dependent manners. This study identified candidate *WRKY* genes with possible roles in abiotic stress and these findings not only contribute to our understanding of WRKY family genes, but also provide valuable information for stress resistance development in *D. catenatum*.

## Introduction

Over their life cycle, plants can suffer various adverse environmental stresses including drought, heat, cold, and high salt. Plants can spontaneously develop a series of response mechanisms when perceiving abiotic stress, such as metabolic reconstruction, cell-tissue remodeling, and gene expression reprogramming. Stress-related transcription factors (TFs) are activated after receiving stress signals, acting as molecular switches to regulate the expression of their target genes by interacting with the *cis*-elements in the gene promoters (Singh et al., 2002), and then participate in the response to abiotic stress (Zhu, 2016). Most plant TFs, such as WRKY, MYB, NAC, bZIP, bHLH, and AP2/DREB, belong to a large gene family. One of the most important and largest transcription factor families in plants is WRKY (Eulgem et al., 2000), which plays a crucial role in many metabolic regulation processes (Rushton et al., 2010).

The WRKY proteins are made up of about 60 amino acids, containing one or two highly conserved WRKYGQK (Trp-Arg-Lys-Tyr-Gly-Gln-Lys) heptapeptides at the N-terminus, and one or two zinc finger structures, C_2_H_2_ (C-X_4-5_-C-X_22-23_-H-X-H) or C_2_HC (C-X_7_-C-X_23_-H-X-C), at the C-terminal (Eulgem et al., 2000; Rushton et al., 2010). The WRKY gene family is divided into three main groups according to the number of conserved WRKY domains and zinc finger structures (Eulgem et al., 2000). Group I contains two WRKY conserved domains and a C_2_H_2_-type zinc finger motif. Group II contains one WRKY conserved domain and a C_2_H_2_-type zinc finger motif. Group III contains one WRKY conserved domain, and a C_2_HC-type zinc finger motif. According to the sequence characteristics of the DNA binding domains in the WRKY proteins, Group II has been further divided into five subgroups, namely IIa, IIb, IIc, IId, and IIe (Eulgem et al., 2000; Rushton et al., 2010). The WRKY TFs can specifically recognize and bind to the W-box (C/TTGACT/C) region of the target gene promoter (Ulker and Somssich, 2004; Bakshi and Oelmüller, 2014), and then activate or inhibit the gene expression at the transcriptional level.

The first *WRKY* gene (*SPF1*) was isolated and characterized from sweet potato (Ishiguro and Nakamura, 1994). Since then, the *WRKY* genes were subsequently reported in other species including lower green algae, terrestrial mosses, and higher land plants (Ulker and Somssich, 2004). The reported *WRKY* genes is distributed as follows: 74 *WRKY* genes in *Arabidopsi*s (Ulker and Somssich, 2004), 103 *WRKY* genes in rice (Ramamoorthy et al., 2008), 55 *WRKY* genes in cucumber (Ling et al., 2011), 102 *WRKY* genes in *G. hirsutum* (Dou et al., 2014), 103 *WRKY* genes in pear (Huang et al., 2015), 71 *WRKY* genes in pepper (Diao et al., 2016), 171 *WRKY* genes in wheat (Ning et al., 2017), 56 *WRKY* genes in melon (Jiao et al., 2018), and 94 *WRKY* genes in sorghum (Baillo et al., 2020). Studies have shown that *WRKY* genes are induced by salt stress and low temperature in *Eucalyptus grandis*, and upregulated under brassinosteroid (BR), salicylic acid (SA), and methyl jasmonate (MeJA) treatments, indicating that WRKY is involved in response to abiotic stress, as well as in hormone signaling pathways in plants (Fan et al., 2018). Most *WRKY* genes in *Populus* were induced by SA and MeJA (Jiang et al., 2014). The expression level of sugarcane *ScWRKY3* was increased under salt, PEG and ABA treatments, but decreased under SA and MeJA treatments (Wang C.-T. et al., 2018). *ScWRKY5* was induced by salt, PEG, SA and ABA (Wang D. et al., 2020). Chen et al. (2012) found that *WRKY* expression was induced under abiotic stress such as drought, saline-alkali, high osmotic stress and high temperature. Studies also showed that TaWRKY1 and TaWRKY33 in wheat (He et al., 2016), and ZmWRKY40 in maize (Wang L. et al., 2018) could enhance drought tolerance of *Arabidopsis* plants. Furthermore, overexpression of *HbWRKY82* enhanced drought and salt tolerance and reduced sensitivity to ABA in transgenic *Arabidopsis thaliana* (Kang et al., 2021), and overexpression of *AhWRKY75* enhanced salt tolerance in transgenic peanut (Zhu et al., 2021).

*Dendrobium catenatum* is a perennial herb of *Dendrobium* orchidaceae with great economic value. As a traditional Chinese medicine, it has immune enhancement activity. Wild *D. catenatum* plants mostly grow on shady mountain rocks or forest trunks, and is often threatened by adversity such as periodic water shortage (Zotz and Winkler, 2013; Wan et al., 2018). Therefore, it is necessary to identify stress-related genes in *D. catenatum* genome and explore their functions. In this study, the *WRKY* gene family was identified and characterized based on the full genomic sequence of *D. catenatum* (Zhang et al., 2016). The features of the *WRKY* gene family were characterized using bioinformatics methods. Meanwhile, the expression of *WRKY* gene members in different tissues and under abiotic stresses as well as hormone treatments was analyzed based on RNA-seq data or using a RT-qPCR assay. This study lays a foundation for future research into the functions of *D. catenatum WRKY* genes.

## Materials and Methods

### Identification of WRKY Family Genes in *Dendrobium catenatum*

The generic feature format (gff), complete genome, proteome, and coding sequence (CDS) files of *D. catenatum* were downloaded from the GenBank database (PRJNA262478, Zhang et al., 2016). The WRKY_domain HMM (Hidden Markov Model) profile (PF03106) was downloaded from the Protein family (Pfam 34.0; http://pfam.xfam.org/) database and used as a query to search the *D. catenatum* protein database using the Bio-linux bioinformatics documentation system to identify putative *WRKY* genes. Candidate sequences were selected with an E value less than e^−10^ (Diao et al., 2016) and then submitted to the Pfam, Conserved Domain Database (CDD, v3.19; https://www.ncbi.nlm.nih.gov/cdd/) and the Simple Modular Architecture Research Tool (SMART, v9; http://smart.embl.de/smart/batch.pl) database to validate the WRKY domains. After removing redundant and incomplete sequences manually, the DcWRKY proteins were finally confirmed. The features including gene locations in the scaffold, isoelectric point (pI), and molecular weight (MW) of DcWRKYs were analyzed using ExPASy-ProtParam (Expasy 3.0; http://web.expasy.org/protparam/). The number of transmembrane regions was determined using TMHMM software (http://www.cbs.dtu.dk/services/TMHMM/). The in-silico subcellular localization of WRKY proteins was predicted using the PSORT tool (https://www.genscript.com/psort.html). The putative *Arabidopsis thaliana* and rice (*Oryza sativa*) *WRKY* members were identified using the same screening method from the Plant Transcription Factor Database (PlantTFDB, v5.0; http://planttfdb.gao-lab.org/tf.php?sp=Ppe&did=Prupe.I004500.1.p).

### Phylogenetic Analysis and Classification of WRKY Proteins

In order to study the evolutionary relationships of WRKY proteins, 213 sequences from three species, *Dendrobium catenatum* (62), *Arabidopsis thaliana* (72), and *Oryza sativa* (79) (Gene ID seen in Supplementary Table S1) were aligned using ClustalW (Thompson et al., 1994), and then a phylogenetic tree was constructed using the Maximum Likelihood (ML) method in MEGA-X (Kumar et al., 2018) with default parameters: poisson model, pairwise deletion, and 1,000 bootstrap replications. WRKY proteins from different subfamilies of the *A. thaliana* and *O. sativa* WRKY families were used as grouping markers. The phylogenetic tree was visualized and enhanced using the EvolView online tool (Evolview v3; https://evolgenius.info/evolview-v2). DNAMAN software (version 6.0.3.99) was used to mark out the structure features of DcWRKY proteins. The sequence logos for Group I, Group II, and Group III of the DcWRKYs were generated using WebLogo (http://weblogo.berkeley.edu/logo.cgi) with default settings.

### Characterization of Conserved Motif, Gene Structure, and Putative *cis*-regulatory Elements

The Multiple Em for Motif Elicitation (MEME, v5.3.3; http://meme-suite.org/tools/meme) program was used to analyze the conserved motifs of the WRKY protein in *D. catenatum*, with the following parameters: number of repetitions-any, maximum number of motifs-10, and the optimum motif widths set from 6 to 100 amino acid residues. The gene exon-intron information for *WRKYs* was extracted from the *D. catenatum* gff file using the TBtools software (Chen et al., 2020). Then, the conserved motifs and gene structures were visualized with TBtools.

To identify potential *cis*-elements in the promoters of *WRKY* genes, 2000-bp sequences upstream of the coding regions of the *DcWRKY* genes were first obtained using the TBtools, and then submitted to the PlantCARE database (http://bioinformatics.psb.ugent.be/webtools/plantcare/html/). Finally, the *cis*-elements were analyzed and visualized by TBtools (Chen et al., 2020).

### Plant Materials, Growth Conditions, and Treatments

*D. catenatum* “Guangnan” tissue culture seedlings were grown under a 12 h/25°C day and 12 h/22°C night regime with a relative humidity of 70% in a growth chamber at Hainan University. The three-month-old plantlets with uniform and robust growth were then selected for subsequent experiments. The seedlings were irrigated with 1/2 MS medium supplemented with 20% PEG8000 and 200 mM NaCl to simulate drought and salt stress, respectively (Zhang et al., 2021). For temperature stress, the plantlets were transferred to a growth chamber set to 42 and 4°C for heat and cold stress, respectively (Zhang et al., 2021). For hormone treatment, the seedlings were irrigated with 1/2 MS medium supplemented with 100 μM ABA or 20 μM SA (Li et al., 2020). The seedlings were then collected and frozen in liquid nitrogen at different time points (0, 3, 6, 9, 12, 24, and 48 h) after treatment.

In addition, the tissues, including roots, stems, leaves, capsules, sepals, petals, lips, gynostemia, and flower stalk were collected from mature plants to analyze the tissue specificity of the *WRKY* genes. The samples were frozen in liquid nitrogen immediately and stored at −80°C. Each treatment was performed with three independent biological replicates, and the samples collected were from five plants for each treatment at each replication.

### Gene Expression Analysis

To examine the expression profiles of the *DcWRKY* genes under drought stress, the RNA-seq data from the SRA website (https://www.ncbi.nlm.nih.gov/sra) (SRP132541) were downloaded and analyzed (Wan et al., 2018). The reference genome index of *D. catenatum* was established by kallisto tools (Bray et al., 2016), from which the expression data were then quantified. Transcripts Per Million (TPM) expression values of the *DcWRKY* genes were log2-transformed. A heat map of the expression profiles of the *DcWRKY* genes was constructed with TBtools. To further study their responses to drought stress, 15 differentially expressed genes across all of the subgroups were selected to study by real-time quantitative PCR (RT-qPCR). Total RNA extraction and RT-qPCR were performed as described by Zhang et al. (2021).

RT-qPCR was used to study the expression levels of the above selected *WRKY* genes under heat, cold, salt stress as well as ABA and SA treatments. Furthermore, the spatial expression patterns were also investigated. Three replicate biological experiments were conducted. Primers are listed in Supplementary Table S2.

### Statistical Analysis

The relative expression levels of *DcWRKY* genes were calculated by the 2^−△△CT^ method (Zhang et al., 2021). All data were calculated using the expression levels under different stresses divided by that under normal condition at the same time points and are presented as the means ± standard error (SE) of three replicates and differences were detected using the Student’s t-test. Asterisks (* or **) indicate a significant difference at *p* < 0.05 or 0.01, respectively.

## Results

### Identification of WRKY Family Members in *D. catenatum*

The WRKY domain model (PF03106) was used as a query to search for WRKY proteins in *D. catenatum* protein files. After removing the redundant and incomplete sequences and further confirming in the SMART, Pfam, and CDD databases, a total of 62 putative *WRKY* genes were obtained and named as DcWRKY1 to DcWRKY62 (Supplementary Table S3) according to their relationships with *Arabidopsis thaliana* WRKY proteins. Sequences including genomic sequences, CDS sequences and protein sequences of *WRKY* genes are listed in Supplementary Table S4. The average length of the DcWRKY proteins was 339 amino acids (ranging from 112 to 717 amino acids). The deduced MWs of the WRKY proteins ranged from 12.86 kDa in DcWRKY37 to 78.36 kDa in DcWRKY23, with an average of 37.50 kDa. Additionally, the theoretical pI ranged from 4.54 (DcWRKY49) to 10.55 (DcWRKY52), with an average of 7.17 (Supplementary Table S3). All of the proteins lacked transmembrane domains, illustrating that they were non-membrane proteins. The DcWRKY proteins were predicted to be nuclear proteins using the PSORT tool. The features of the DcWRKY proteins suggest that there are significant differences among them, which may reflect a diversity of functions in *D. catenatum*.

### Phylogenetic Classification of DcWRKYs

**FIGURE 1:**
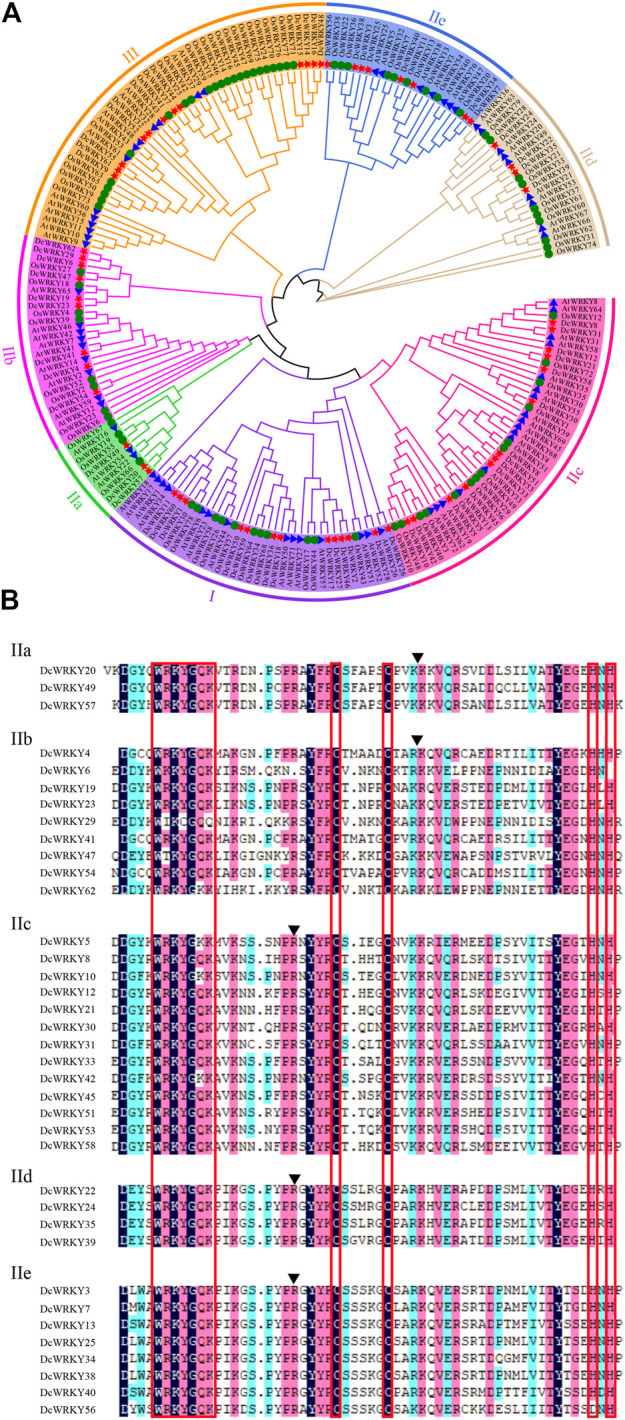
Comparison of the WRKY family in plants. **(A)** Phylogenetic analyses of WRKY proteins from *D. catenatum*, *Arabidopsis*, and rice. A phylogenetic tree of WRKY proteins was constructed using MEGA-X with default parameters. The subgroups are indicated with different colors. The red stars represent *D. catenatum* WRKYs (DcWRKYs), the blue triangles represent *Arabidopsis thaliana* WRKYs (AtWRKYs), and the green circles represent *Oryza sativa* WRKYs (OsWRKYs). **(B)** Multiple sequence alignments of the WRKY domains from the group II DcWRKY members. Alignment was performed using DNAMAN. The red box indicates the conserved WRKY amino acid sequence and zinc-finger domain. For each subgroup, the position of a conserved intron is indicated by an arrowhead.

In order to evaluate the evolutionary relationships of the *WRKY* gene family in *D. catenatum*, a combined ML tree was constructed using MEGA-X software. The phylogenetic tree showed that the 213 WRKYs (Supplementary Table S1) were divided into three subfamilies: group I, group II, and group III (Figure 1). Group II, the largest group, contained 37 DcWRKYs and accounted for 60% of all DcWRKYs in *D. catenatum*. Groups I and III had 13 and 12 DcWRKY members, respectively (Figure 1A, Supplementary Table S3). As shown in Figure 1A and Supplementary Figure S1, a similar member distribution in each subgroup was also found in *A. thaliana* and *O. sativa*, indicative of similar evolutionary trajectories for the *WRKY* genes in the three species.

All of the proteins contained the WRKYGQK heptapeptide, which was considered the hallmark of WRKY family (Supplementary Figure S2). Group I had two conserved WRKY domains located in the N- and C-termini of the protein as well as the zinc-finger motifs C-X_4_-C-X_22_-H-X_1_-H (Supplementary Figure S2A). Group II and Group III both had only one WRKY domain. Group II had the zinc-finger motif C-X_5_-C-X_23_-H-X_1_-H (Supplementary Figure S2B), while group III had the zinc-finger domain C-X_7_-C-X_23_-H-X_1_-C (Supplementary Figure S2C). Moreover, the DcWRKY proteins in group II could be further classified into five subgroups (IIa, IIb, IIc, IId, and IIe) containing 3, 9, 13, 4, and 8 members, respectively (Figure 1A, Supplementary Table S3). It’s worth noting that among all the groups and subgroups, the most DcWRKY members were present in subgroup IIc, similar to AtWRKYs (Supplementary Figure S1). As shown in Figure 1B, two types of intron structures were present in the conserved regions of the DcWRKY domains. Among them, one was a PR intron present in subgroups IIc, IId, and IIe, which was spliced at the codon of the R amino acid between the WRKYGQK heptapeptide and zinc-finger motif; the other was the VQR intron present in subgroups IIa and IIb, which was located within the zinc-finger structure (C-X_5_-C-X_5_-VQR-X_15_-H-X_1_-H).

### Conserved Motifs and Gene Structures of MYB Family in *D. catenatum*

**FIGURE 2:**
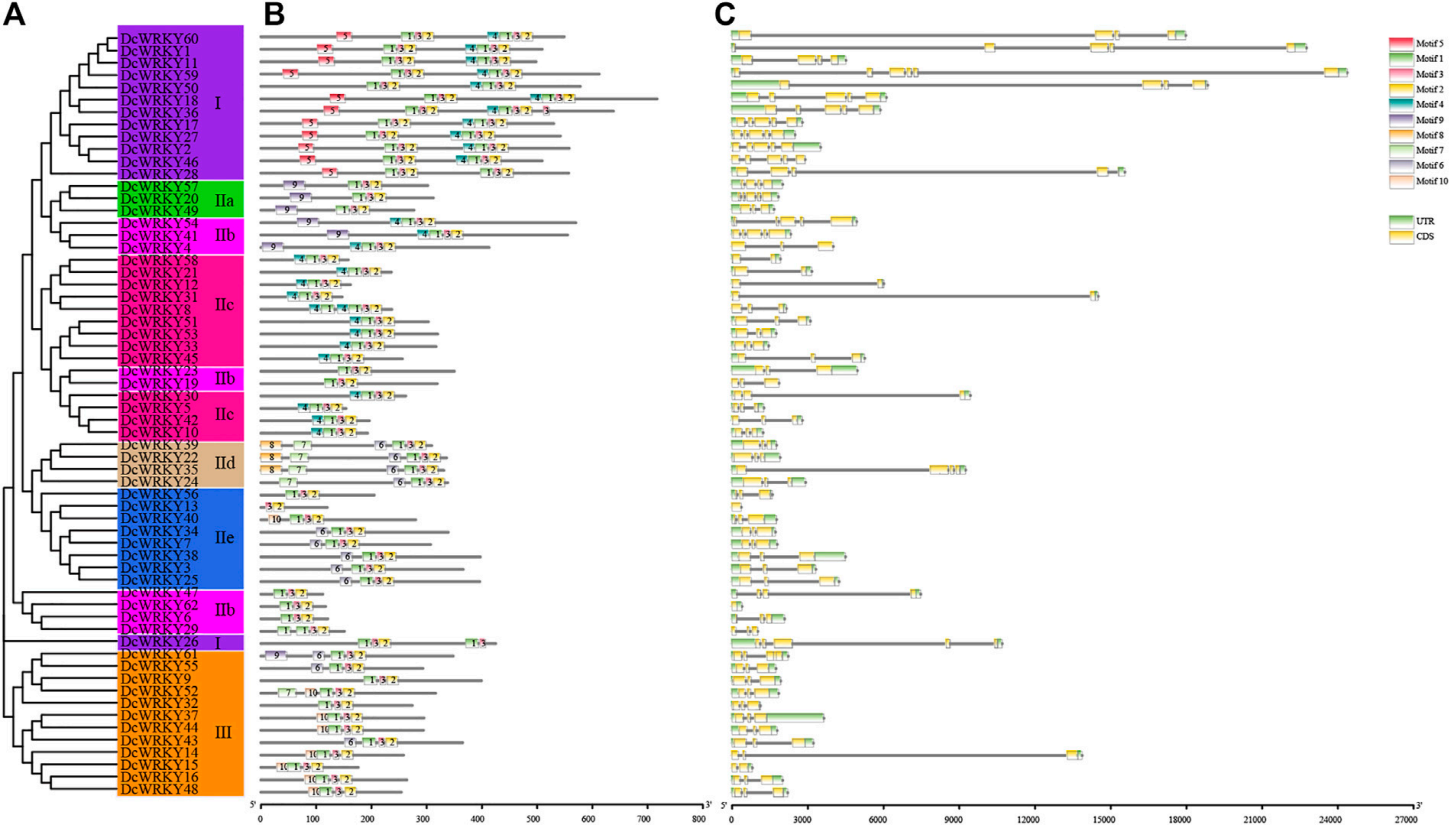
Phylogenetic relationships, motifs, and structures of DcWRKY family members. **(A)** A phylogenetic tree of 62 DcWRKY proteins was constructed with the Maximum Likelihood method. The different subgroups are indicated with different background colors and letters. **(B)** Conserved motifs of DcWRKY proteins. Different motifs are represented by various colored boxes and different numbers. **(C)** Exon/intron structures of *DcWRKY* genes. Exon(s), intron(s), and UTR(s) are represented by yellow boxes, black lines, and green boxes, respectively. The phylogenetic tree, conserved motifs, and gene structures were predicted with TBtools.

In order to understand the conservation and diversification of DcWRKYs, the putative motifs of all DcWRKY proteins were predicted by MEME motif analysis. A total of 10 distinct motifs, named motif 1 to motif 10, were detected (Supplementary Figure S3). The lengths of these conserved motifs varied from 11 (motif 3) to 39 (motif 8 and motif 9) amino acids. The number of the conserved motifs for each DcWRKY protein ranged from 3 to 8 (Figure 2B). All the DcWRKYs contained motif 1-3-2, consisting of the WRKY domain and zinc-finger motif (Supplementary Figures S2, S3). As expected, the DcWRKYs that were categorized into the same group or subgroup shared highly similar motif compositions (Figure 2B, Supplementary Table S5). For instance, the DcWRKY proteins from group I contained motif 5-1-3-2-4-1-3-2 except DcWRKY 26, which contained motif 1-3-2-1-3; the members from subgroup IIa all contained motif 9-1-3-2. Motif 5 was only found in group I, and motif 8 was only found in subgroup IId, suggesting that these two motifs might have specific roles.

The exon/intron distributions and the intron numbers were analyzed to further detect structural features and evolutionary events of the *DcWRKY* genes. The number of introns in the *DcWRKY* family varied from 0 to 5. A total of 35 (56%) *DcWRKY* genes with two introns accounted for the largest proportion, followed by 12 (19%), 6 (10%), 5 (8%), 2 (3%), and 2 (3%) genes, possessing 4, 3, 1, 5, and 0 introns, respectively (Figure 2C, Supplementary Table S5). Meanwhile, *DcWRKY* genes belonging to the same subfamily shared a similar exon/intron structure. For example, *DcWRKYs* in subgroup IIe contained 0–2 introns, while approximately 88% (7/8) possessed two introns. *DcWRKYs* in subgroup IIc contained one to two introns, while approximately 69% (9/13) possessed two introns. *DcWRKYs* in group I contained three to five introns, while approximately 62% (8/13) possessed four introns.

Overall, the closely related DcWRKYs in the phylogenetic tree shared similar common motif compositions and gene structures, suggesting that the DcWRKYs within the same group/subgroup may play similar functional roles.

### Promoter Analysis of *DcWRKY* Genes

**FIGURE 3:**
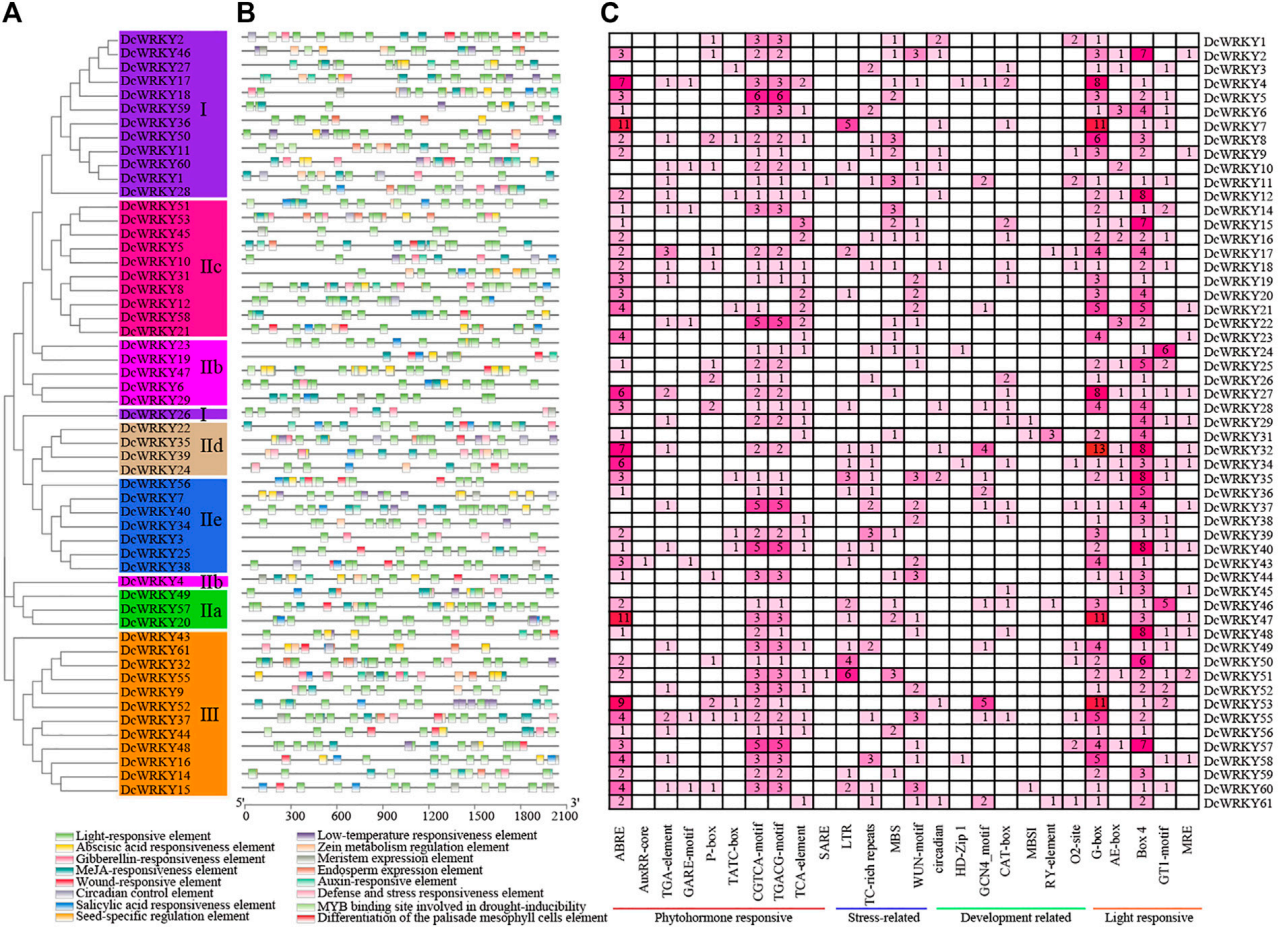
Analysis of *cis*-elements in the *DcWRKY* genes promoter regions. **(A)** A phylogenetic tree of 62 DcWRKY proteins was constructed with the ML method. The different subgroups are indicated with different background colors and letters. **(B)** The different colored blocks represent the different types of *cis*-elements and their locations in each *DcWRKY* gene. **(C)** The different colors and numbers in the grid indicate the numbers of different promoter elements in the *DcWRKY* genes. The types, numbers, and locations of potential elements in the promoter regions 2-kb upstream of the *DcWRKY* genes were analyzed by PlantCARE.

To understand the roles of *cis*-regulatory elements in *DcWRKYs*, the *cis*-elements were identified in the 2 kb upstream sequence from the translation start site (ATG) of each *DcWRKY* gene using PlantCARE software. In this study, various *cis*-elements were found in 55 out of 62 *DcWRKY* genes (Figure 3, Supplementary Table S6), while the remaining seven *WRKYs* could not be detected because of a short sequence upstream of ATG. All of the detected *cis*-elements could be classified into four types according to their functions: 1) phytohormone responsive, 2) abiotic and biotic stress-responsive, 3) development-related, and 4) light-responsive elements. 1) The phytohormone responsive *cis*-acting elements, including abscisic acid responsiveness (ABRE), auxin responsiveness (AuxRR-core and TGA-element), gibberellin-responsive elements (GARE-motif, P-box, and TATC-box), MeJA-responsive (CGTCA-motif and TGACG-motif), and salicylic acid-responsive (TCA-element and SARE), were widely present in the promoter region. Among these elements, the ABA and auxin-related elements (ABRE, CGTCA-motif and TGACG-motif) accounted for the largest part, while the SARE element was only found in the promoter region of *DcWRKY21* and *DcWRKY51*, suggesting that the two genes might function in the SA signaling pathway. 2) Four stress-related *cis-*elements including low-temperature responsiveness (LTR), defense and stress responsiveness (TC-rich repeats), drought-inducibility (MBS), and wound-responsive element (WUN-motif) were detected in the *WRKY* promoter regions. The results showed that LTR was detected in 18 (33%) *WRKY* gene promoters, TC-rich repeats elements were detected in 21 (38%) *WRKY* gene promoters, MBS was detected in 23 (42%) *WRKY* gene promoters, and WUN-motif was detected in 25 (46%) gene promoters. Moreover, 29 (53%) gene promoters contained more than two stress-related elements, suggesting that the DcWRKYs may play roles in multiple stress responses. 3) The third type is plant development-related elements, which were distributed sporadically in the promoter regions. MBSI, a MYB binding site involved in regulation of flavonoid biosynthetic genes, only existed in *DcWRKY29, 31,* and *60*, suggesting that these three genes may regulate flavonoid metabolism. 4) The largest number of *cis-*elements observed across the 62 *DcWRKY* genes, was the type associated with light-related responsiveness, such as G-box, Box 4, AE-box and GT1-motif. G-box was detected in almost all the *DcWRKYs* promoter regions except *DcWRKY10, 22, 29, 36, 45*, and *48.*

### Expression Profiles of *WRKY* Genes Under Drought Stress Based on RNA-Seq and RT-qPCR

**FIGURE 4:**
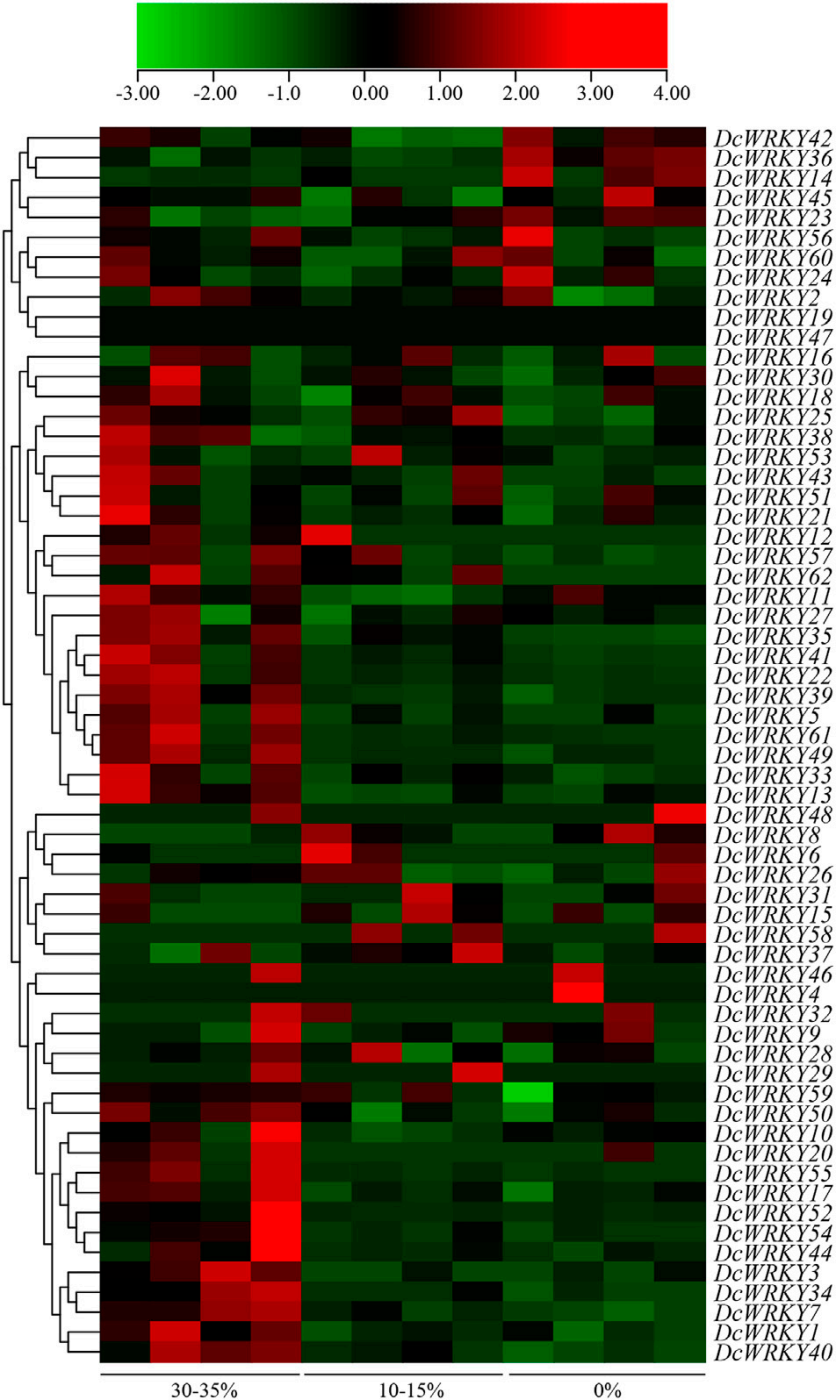
Heat map of *DcWRKY* genes under different volumetric water contents of the base material. Data were normalized relative to each gene’s mean expression value across all treatments and log2-transformed. TPM values were used to create heat map showing the expression of *DcWRKY* genes. The expression level ranges from low expression (green) to high expression (red).

To investigate the responses of *DcWRKY* genes to drought stress, the RNA-seq data (SRP132541) were downloaded and analyzed, and the TPM of each *WRKY* gene was determined based on four replicates (Supplementary Table S7). Overall, most *DcWRKY* genes showed differential expression patterns when the volumetric water content of the base material decreased from 30–35% to 0% (Figure 4), which indicated that a high number of *DcWRKY* genes were responsive to drought stress.

**FIGURE 5:**
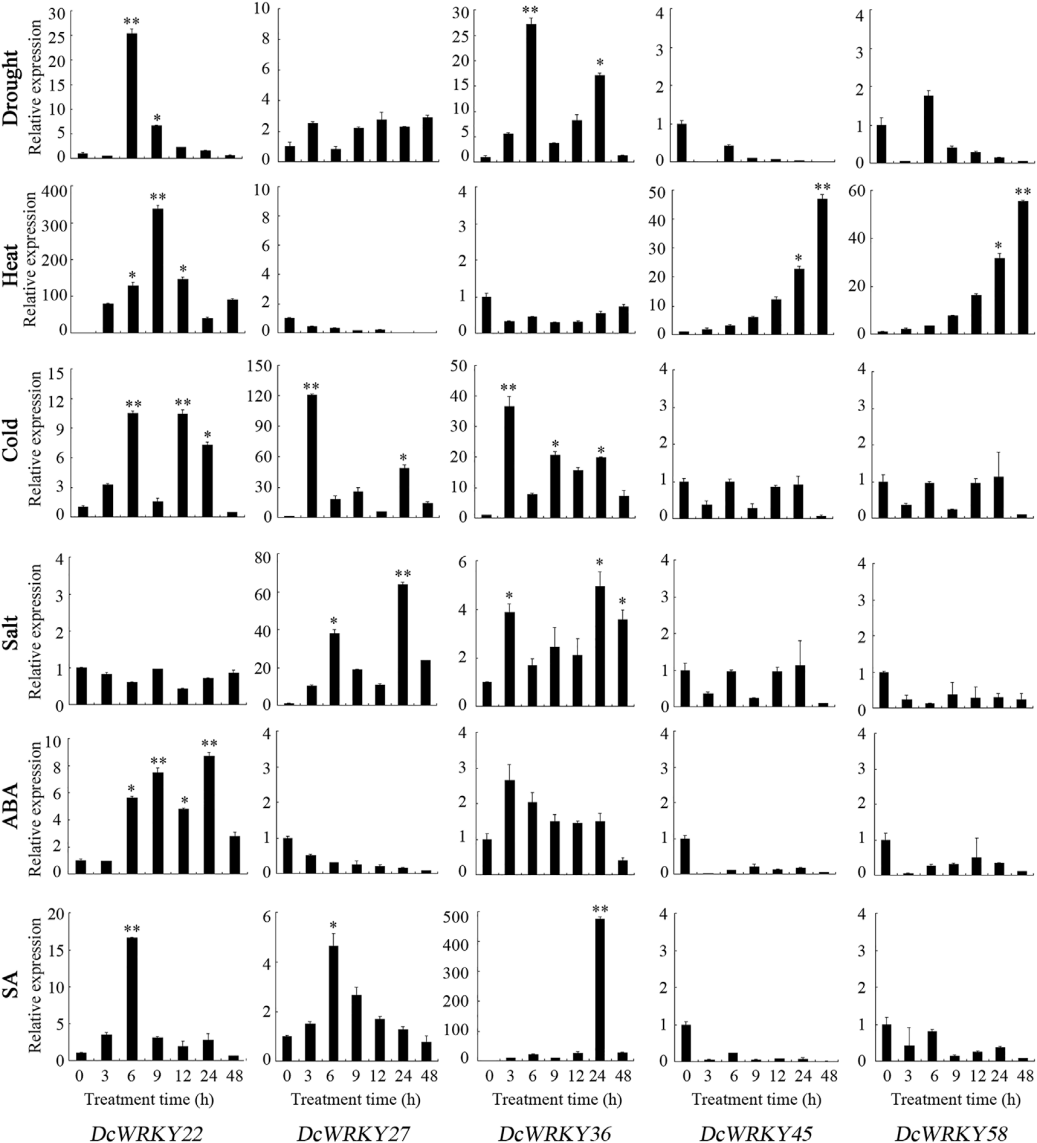
Real-time quantitative PCR analysis of 5 *DcWRKY* genes under abiotic stress and hormone treatment in roots. Vertical bars indicate the standard deviation (*n* = 3). Values of 0, 3, 6, 9, 12, 24, and 48 indicate hours after treatment. The unstressed level (0 h) was used as a control. Asterisks (∗ or ∗∗) indicate a significant difference at *p* < 0.05 or 0.01, respectively.

**FIGURE 6:**
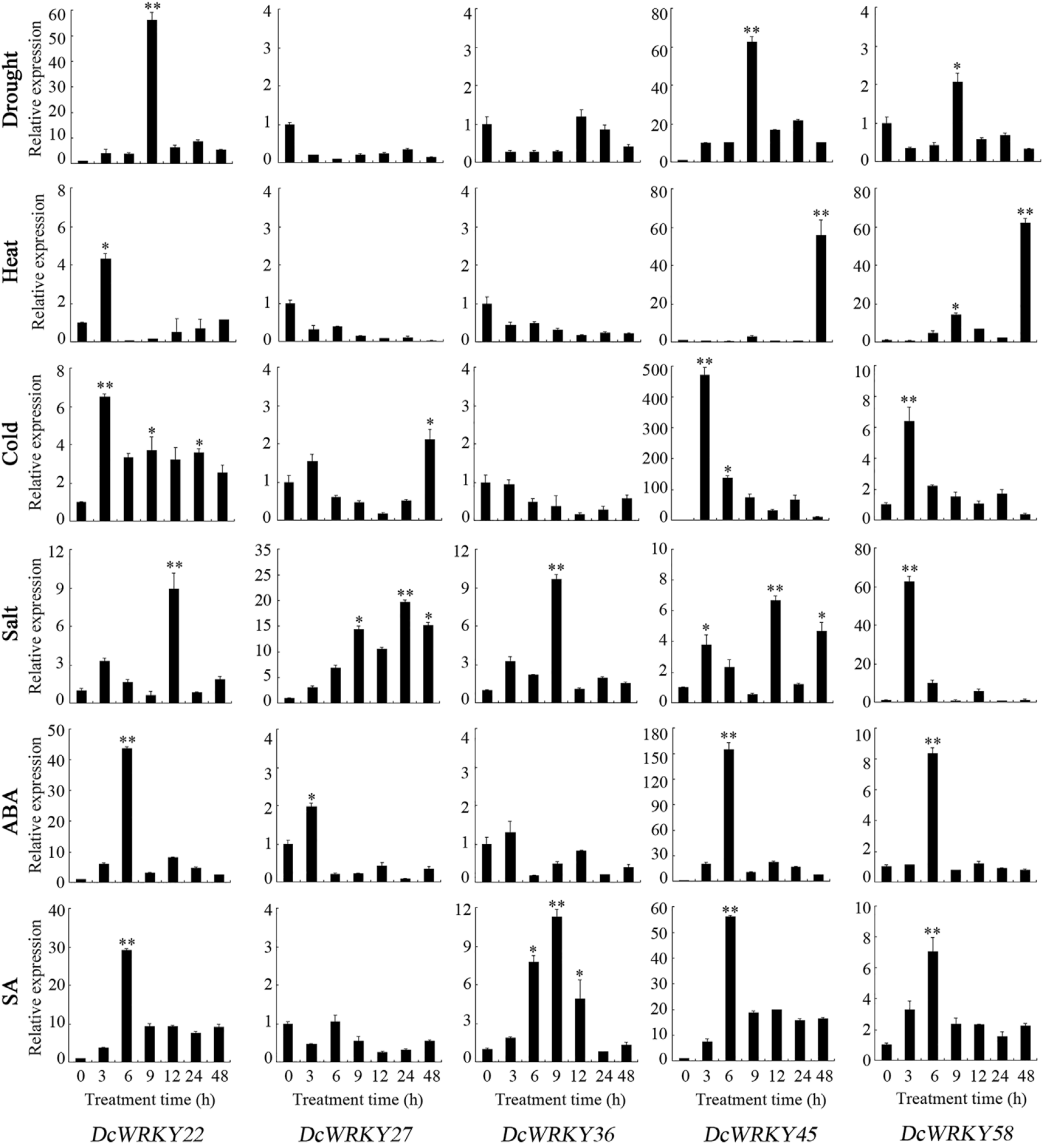
Real-time quantitative PCR analysis of 5 *DcWRKY* genes under abiotic stress and hormone treatment in leaves. Vertical bars indicate the standard deviation (*n* = 3). Values of 0, 3, 6, 9, 12, 24, and 48 indicate hours after treatment. The unstressed level (0 h) was used as a control. Asterisks (∗ or ∗∗) indicate a significant difference at *p* < 0.05 or 0.01, respectively.

To further explore their responses to drought stress, 15 *WRKY* genes across all of the subgroups were selected according to the RNA-seq data, and their expression patterns were analyzed using RT-qPCR after exposure to 20% PEG8000. In roots, *DcWRKY22*, *DcWRKY36*, and *DcWRKY41* were highly induced by 20-fold after PEG treatment, among which *DcWRKY36* exhibited the highest expression level (27-fold increase) after treatment for 6 h (Figure 5, Supplementary Figure S4). While in leaves, *DcWRKY22* and *DcWRKY45* were highly induced by PEG stress (Figure 6, Supplementary Figure S5). Consistently, the expression of both the two genes reached the peak after treatment for 9 h, among which *DcWRKY45* had the highest expression level (63-fold increase).

### Expression Patterns of Selected *DcWRKY* Genes Under Other Abiotic Stresses

In order to investigate the expression of these *DcWRKY* genes under other abiotic stresses, the *D. catenatum* seedlings were treated with heat, cold and salt. As shown in Figure 5, 6, and Supplementary Figures S4, S5, nine genes were up-regulated, and six genes were down-regulated in roots under heat stress, while five genes were up-regulated in leaves. *DcWRKY22* was highly expressed in both roots and leaves when treated with heat, with a 337-fold increase in roots after treatment for 9 h. Under cold stress, 10 *DcWRKY* genes were up-regulated in roots, among which *DcWRKY27* and *DcWRKY41* were highly induced after short-term heat stress (42°C for 3 h), with the expression levels 100-fold higher than those under normal condition. *DcWRKY45* was extremely induced by heat stress in leaves and up-regulated by 473-fold after 3 h. Under salinity stress, four *DcWRKY* genes (*DcWRKY27*, *DcWRKY37*, *DcWRKY41* and *DcWRKY42*) were highly induced in roots. In particular, the expression of *DcWRKY27* was up-regulated the most after 24 h of salt treatment. While all the selected genes were up-regulated in leaves, among which *DcWRKY58* had the highest expression level when treated with salinity for 3 h, with a 63-fold increase.

### Expression Patterns of Selected *DcWRKY* Genes Under Hormone Treatments

To identify specific *DcWRKY* genes that are potentially involved in ABA or SA signaling pathways, we examined the transcriptional levels of these 15 genes under treatment with either 100 μM ABA or 20 μM SA by RT-qPCR. Five *DcWRKY* genes including *DcWRKY22*, *DcWRKY23*, *DcWRKY36*, *DcWRKY41* and *DcWRKY49* were up-regulated, nine *DcWRKY* genes including *DcWRKY27*, *DcWRKY35*, *DcWRKY37*, *DcWRKY42*, *DcWRKY45*, *DcWRKY53*, *DcWRKY56*, *DcWRKY57* and *DcWRKY58* were down-regulated in roots under ABA treatment (Figure 5, Supplementary Figure S4). While in leaves, 10 *DcWRKY* genes (except *DcWRKY36*, *DcWRKY37*, *DcWRKY41*, *DcWRKY42* and *DcWRKY49*) were up-regulated (Figure 6, Supplementary Figure S5). When treated with SA, eight *DcWRKY* genes (*DcWRKY22*, *DcWRKY23*, *DcWRKY27*, *DcWRKY35*, *DcWRKY36*, *DcWRKY37*, *DcWRKY41* and *DcWRKY42*) were up-regulated, and six *DcWRKY* genes (*DcWRKY45*, *DcWRKY49*, *DcWRKY53*, *DcWRKY56*, *DcWRKY57* and *DcWRKY58*) were down-regulated in roots (Figure 5, Supplementary Figure S4). However, most tested genes except that *DcWRKY27* and *DcWRKY49* were significantly up-regulated in leaves (Figure 6, Supplementary Figure S5).

### Analysis of Selected *DcWRKY* Genes in Different Tissues by RT-qPCR

**FIGURE 7:**
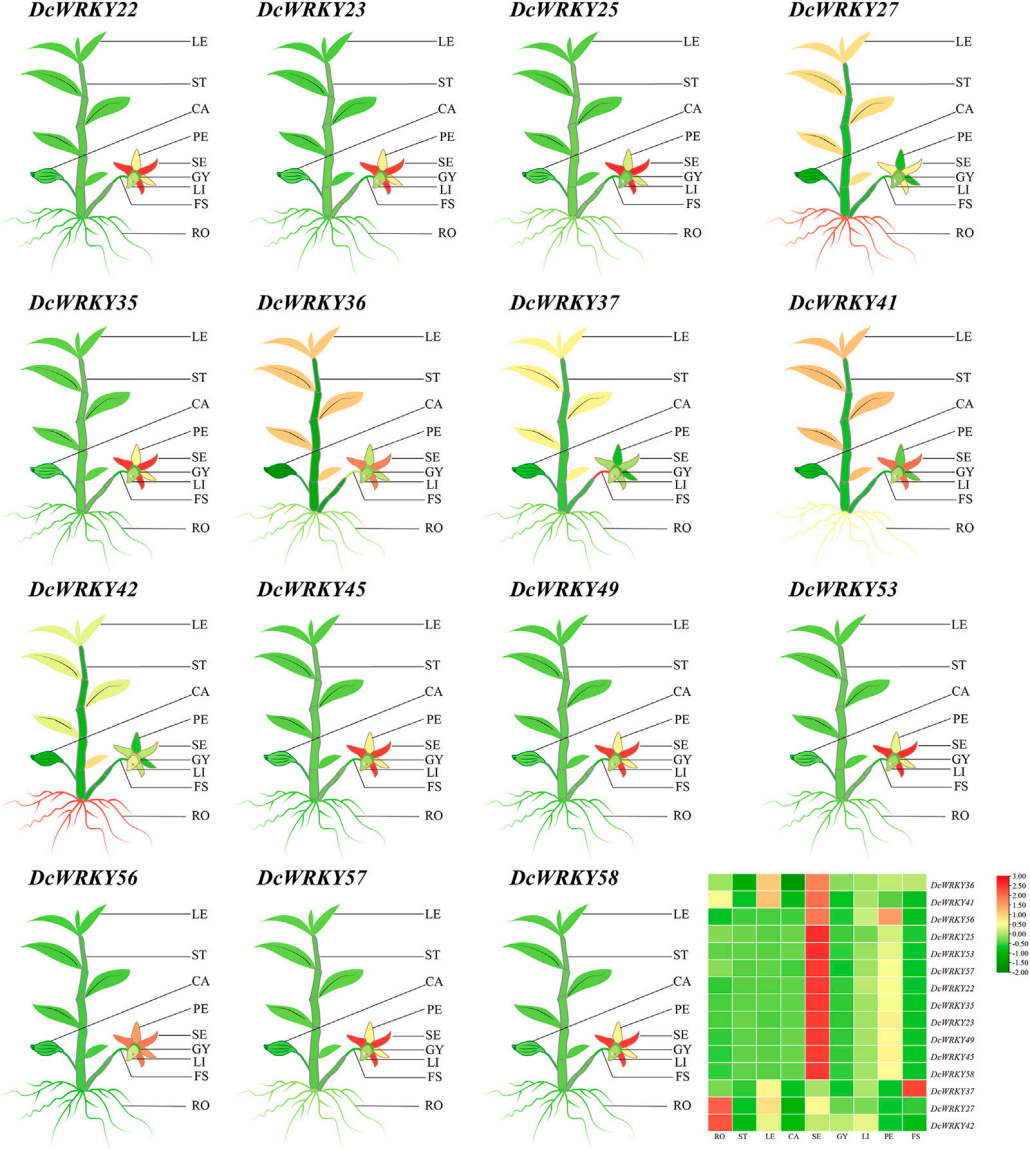
Tissue-specific expression of representative *DcWRKY* genes by RT-qPCR. The mean expression value was calculated from three independent biological replicates relative to that in roots. The mean expression values were visualized by TBtools; red and green represent high and low expression levels, respectively. The relative expression values are provided in Supplementary Table S8. LE, leaf; ST, stem; CA, capsule; PE: petal; SE, sepal; GY, gynostemia; LI, lip; FS, flower stalk; RO, root.

To investigate the spatial expression profiles of the 15 *WRKY* genes in *D. catenatum*, RT-qPCR was used to analyze their expression in nine tissues including roots, stems, leaves, capsules, sepals, petals, lips, gynostemia, and flower stalk. As shown in Figure 7 and Supplementary Table S8, the expression levels of all the selected *WRKY* genes were higher in the reproductive organs including sepals, petals, and lips than in the vegetative organs including roots, stems and leaves.

## Discussion

The WRKY transcription factor family is one of the most important gene families involved in plant development and biotic and abiotic stress response (Chen et al., 2018). The evolutionary relationships and function analysis of *WRKY* genes have been identified in many species, such as *Arabidopsis* (Ulker and Somssich, 2004), rice (Ramamoorthy et al., 2008), wheat (Ning et al., 2017), melon (Jiao et al., 2018), watermelon (Yang et al., 2018), among others. The number of *WRKY* genes ranges from 56 in melon to 171 in wheat, illustrating a high diversity. In this study, we identified 62 *WRKY* genes from the *D. catenatum* genome. Recent studies have shown that gene duplication events, including tandem duplication, segmental duplication, and whole-genome duplication play an important role, not only in the process of genome rearrangement and expansion, but also in the diversification of gene function and production of large numbers of gene families (Cannon et al., 2004). *WRKY* gene duplication events have been found in rice (Ross et al., 2007), tomato (Huang et al., 2012), *Populus trichocarpa* (He et al., 2012), and cotton (Dou et al., 2014), but not in cucumber (Ling et al., 2011). Compared to the small genome size of *Arabidopsis* (125 Mb, 72 *WRKYs* identified in this study) and rice (480 Mb, 79 *WRKYs* identified in this study), the *D. catenatum* genome (1.11 Gb) (Zhang et al., 2016) is substantially larger. However, the number of genes is similar in the three species, suggesting that *WRKY* gene duplication might not occur during gene evolution in *D. catenatum*. Meanwhile, the number of gene family does not completely correlate with the genome size (Sun et al., 2019). In addition, with the improvement of genome sequencing accuracy and assembly, the upgrading and updating of search and analysis software, and the existence of alternative splicing in the genome, it is quite possible to find new WRKY members in the *D. catenatum* genome as well as other species in the future.

According to the classification of WRKY proteins in other plants (Eulgem et al., 2000; Rushton et al., 2010), 62 DcWRKY proteins were also classified into three groups (group I, II, and III) and group II was further classified into five subgroups (subgroup IIa to IIe) based on their conserved WRKY domain and zinc-finger motif. The number of DcWRKYs in each group is similar to that in *Arabidopsis* and *O. sativa* (Figure 1A and Supplementary Figure S1), indicating similar evolutionary patterns in *D. catenatum*, *Arabidopsis*, and *O. sativa*. Phylogenetic analysis showed that group I was the original ancestor of groups II and III during the evolution of the *WRKY* gene family in plants, and that the WRKY gene family further evolved into groups II and III through the preservation and deletion of the WRKY domain at the N-terminus and the change of the WRKY domain at the C-terminus of group I (Zhang and Wang, 2005). In this study, *WRKY* genes in the *D. catenatum* genome were identified and grouped using the structural characteristics of the conserved WRKY domain in the WRKY gene family of known species. Analysis of the WRKY domain structure revealed that the structure of the WRKY gene family in *D. catenatum* is highly conserved. However, DcWRKY62 in subgroup IIb and DcWRKY5, 10, 42 in subgroup IIc contained the WRKY domain WRKYGKK, with a mutation in the sixth amino acid in the conserved heptapeptide (Figure 1B). This variant of the WRKY domain has been found in the genomes of tomato (Huang et al., 2012), apple (Meng et al., 2016), pepper (Diao et al., 2016), and *Camellia sinensis* (Wu et al., 2016). This result suggests that the WRKY gene family is highly conserved in plant structure, although there are small variations in WRKY domain. Meanwhile, the variation of the WRKYGQK heptapeptide in the WRKY domain also indicated the diversity of the *WRKY* gene family.

The intron-exon structures can provide important evidence for gene evolutionary relationships. In this study, we systematically analyzed the structure distribution of *WRKY* gene family members in *D. catenatum*. Through analysis of gene structures, *DcWRKY* gene family members were found to be comprised of 0–5 introns, while the number of introns was 0–8 in rice (Xie et al., 2005) and 0 to 22 in *Musa acuminate* (Goel et al., 2016), respectively. These results suggest that *DcWRKY*s exhibited a low gene structure diversity. Furthermore, we found that *DcWRKY* genes belonging to the same subfamily shared a similar exon-intron structure. For example, approximately 88% of *DcWRKY* members in subgroup IIe possessed two introns, 69% of members in subgroup IIc contained two introns, and 62% of *DcWRKYs* in group I possessed four introns (Figure 2C, Supplementary Table S5), a finding similar to that found in wheat (Ning et al., 2017) and cassava (Wei et al., 2016). These results suggest that the distribution pattern of introns and exons was group-specific.

Increasing evidence has shown that WRKY proteins in various plant species are involved in plant development (Luo et al., 2013; Liu et al., 2015) and response to various abiotic stresses such as drought, cold, and salt (Chen et al., 2012; Singh et al., 2017). In *Arabidopsis* and rice, at least 20 and 54 *WRKY* genes, respectively were identified in response to diverse abiotic stress (Li et al., 2013; Phukan et al., 2016). The RNA-seq results showed that most *WRKY* genes were induced by drought stress (Figure 4). Furthermore, we tested the expression levels of 15 selected genes using RT-qPCR (Figures 5, 6, and Supplementary Figures S4, S5). The results showed that *DcWRKY22*, *DcWRKY36* and *DcWRKY45* were induced by drought stress, consistent with the RNA-seq data. *DcWRKY22* was highly induced by heat stress (Figure 5), and *DcWRKY45* was highly induced by cold stress (Figure 6), indicating that these two genes are potential candidate genes for promoting temperature tolerance using knock-out or transgenic techniques (Zhou et al., 2020). Among all the tested genes, the expression levels of *DcWRKY27* and *DcWRKY58* were the highest under salinity treatment in roots and leaves, respectively, suggesting they might play roles in response to salt stress. *Cis*-acting elements in gene promoters are closely related to the roles of genes in stress and response (Yamaguchi-Shinozaki and Shinozaki, 2005). Many *cis*-acting elements were also detected in the promoters of the *WRKY* genes (Figure 3, Supplementary Table S6). For example, the expression level of *DcWRKY22* gene was up-regulated significantly under drought stress, and the MBS element was detected in its promoter region, suggesting that some proteins regulated gene expression through interacting with the elements. We also analyzed the expression of *DcWRKY* genes in different tissues using RT-qPCR. The results demonstrated that the expression patterns of *DcWRKY* genes were different and tissue-specific. There were higher expression levels of many *DcWRKY* genes in reproductive organs than in vegetative organs (Figure 7), indicating that *D. catenatum* WRKY members may be involved in later growth and development.

As essential endogenous signal molecules in plants, phytohormones can regulate plant growth and development under severe stress conditions (Kermode, 2005; Ryu and Cho, 2015). Numerous studies showed that the expression of *WRKY* genes was induced after hormone treatment (Yang et al., 2009; Jiang et al., 2014). The *DlWRKY* gene was upregulated in longan under SA and MeJA treatment (Jue et al., 2018), the peanut *WRKY1* and *WRKY12* genes were up-regulated with SA and JA treatment (Song et al., 2016), and PlWRKY65 was verified as a positive disease resistance regulator in *Paeonia lactiflora* to regulate JA and SA hormone signaling pathways (Wang X. et al., 2020). By analyzing the *cis*-acting elements in the promoter regions of *WRKY* genes, we found that ABA-, SA-, MeJA-, IAA- and GA-related elements were present in the promoter regions of most *WRKY* genes, indicating that these genes may be involved in hormone signal pathways. As shown in Figures 5, 6, and Supplementary Figures S4, S5, almost all the *DcWRKY* genes were induced by ABA and SA in leaves, while some genes were up-regulated, and other genes were down-regulated when treated with ABA or SA in roots. *DcWRKY22* induction by drought and heat in roots was up-regulated by ABA and SA, *DcWRKY45* induction by drought and cold in leaves was up-regulated by ABA and SA, *DcWRKY36* was highly induced by drought and hormones treatments in leaves, and *DcWRKY58* was highly induced by salt stress and hormones treatments in leaves. In contrast, *DcWRKY27* was up-regulated under salinity in both roots and leaves, but down-regulated in roots when treated with ABA and in leaves when treated with SA, indicating that it may function in response to salt stress in a complicated way. Taken together, these results suggest that these genes may function in response to abiotic stresses in an ABA- and SA-dependent manner.

## Conclusion

**FIGURE 8:**
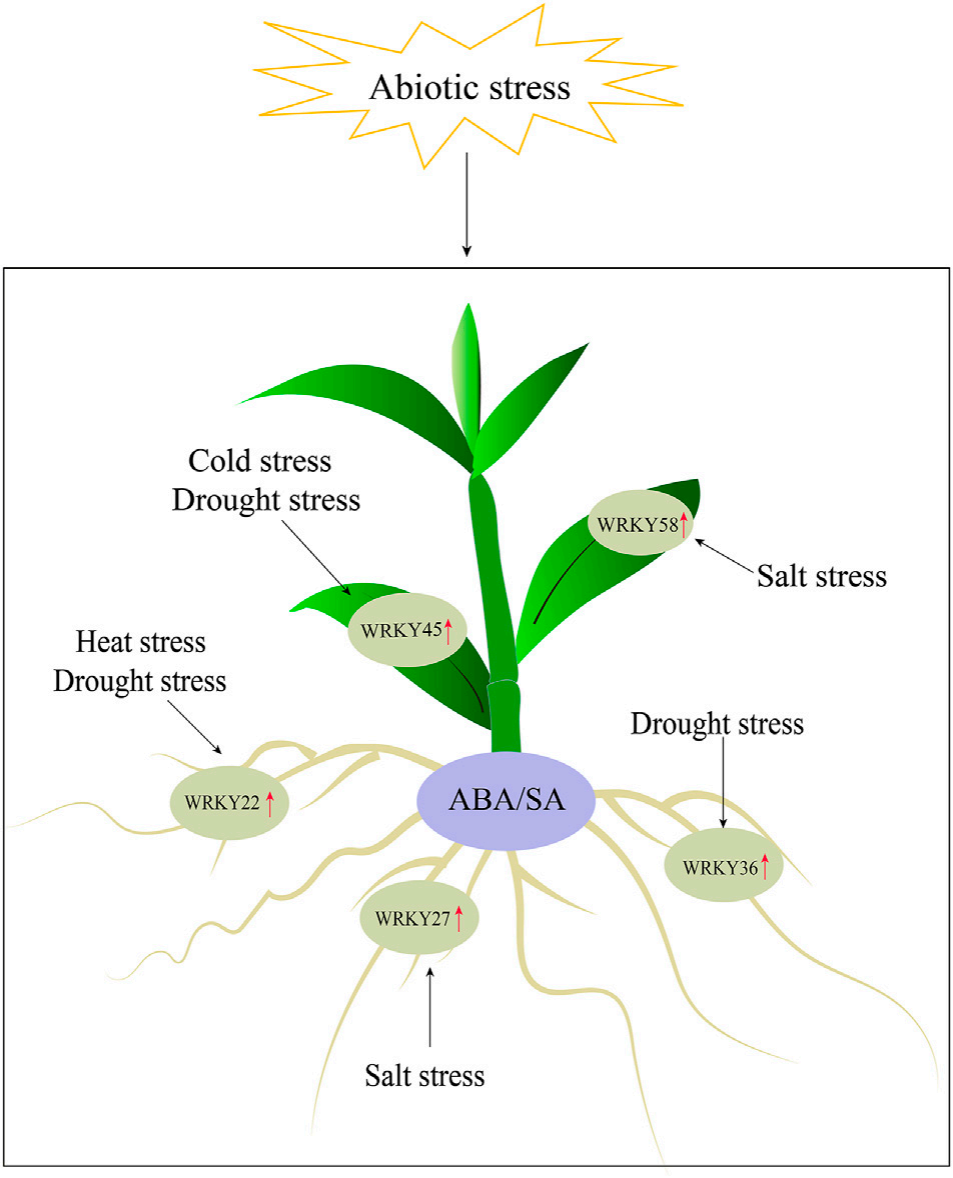
Model of the five *DcWRKY* genes responding to abiotic stress.

In this study, we performed a genome-wide identification of *WRKY* genes in *D. catenatum* and identified a total of 62 *DcWRKY* genes. These genes were classified into three groups with group II further classified into five subgroups based on their phylogenetic relationships. The basic features, gene structures, conserved motifs, and *cis*-elements of these genes were analyzed, providing a foundational understanding of the evolutionary relationships within the *DcWRKY* gene family. The expression of *DcWRKY* genes was studied using RNA-seq and RT-qPCR, and the results revealed that 15 selected *DcWRKY* genes were tissue-specific and influenced by abiotic stresses. Furthermore, we screened five genes (*DcWRKY22*, *DcWRKY27*, *DcWRKY36*, *DcWRKY45*, and *DcWRKY58*) expressed significantly in response to abiotic stresses, and functioned in an ABA- and SA-dependent manner (Figure 8). Our results presented here provide a basis for functional characterization of *DcWRKY* genes involved in stress resistance in *D. catenatum*.

## Data Availability

The original contributions presented in the study are included in the article/Supplementary Material, further inquiries can be directed to the corresponding authors.

## Abbreviations

ABA, Abscisic acid; BR, Brassinosteroid; *D. catenatum*, *Dendrobium catenatum;* MeJA, Methyl jasmonate; ML, Maximum Likelihood; MW, Molecular weight; pI, Isoelectric point; RT-qPCR, Quantitative real-time polymerase chain reaction; SA, Salicylic acid; TF, Transcription factor; TPM, Transcripts Per Million; WT, Wild type.

## References

[B1] Baillo E. H. Hanif M. S. Guo Y. Zhang Z. Xu P. Algam S. A. (2020). Genome-wide Identification of WRKY Transcription Factor Family Members in Sorghum (*Sorghum Bicolor* (L.) Moench). PLoS ONE 15 (8), e0236651. 10.1371/journal.pone.0236651 32804948 PMC7430707

[B2] Bakshi M. Oelmüller R. (2014). WRKY Transcription Factors. Plant Signaling Behav. 9, e27700. 10.4161/psb.27700 PMC4091213 24492469

[B3] Bray N. L. Pimentel H. Melsted P. Pachter L. (2016). Near-optimal Probabilistic RNA-Seq Quantification. Nat. Biotechnol. 34, 525–527. 10.1038/nbt.3519 27043002

[B4] Cannon S. B. Mitra A. Baumgarten A. Young N. D. May G. (2004). The Roles of Segmental and Tandem Gene Duplication in the Evolution of Large Gene Families in *Arabidopsis thaliana* . BMC Plant Biol. 4, 10. 10.1186/1471-2229-4-10 15171794 PMC446195

[B5] Chen C. Chen H. Zhang Y. Thomas H. R. Frank M. H. He Y. (2020). TBtools: An Integrative Toolkit Developed for Interactive Analyses of Big Biological Data. Mol. Plant 13, 1194–1202. 10.1016/j.molp.2020.06.009 32585190

[B6] Chen F. Hu Y. Vannozzi A. Wu K. Cai H. Qin Y. (2018). The WRKY Transcription Factor Family in Model Plants and Crops. Crit. Rev. Plant Sci. 36 (5), 311–335. 10.1080/07352689.2018.1441103

[B7] Chen L. Song Y. Li S. Zhang L. Zou C. Yu D. (2012). The Role of WRKY Transcription Factors in Plant Abiotic Stresses. Biochim. Biophys. Acta (Bba) - Gene Regul. Mech. 1819 (2), 120–128. 10.1016/j.bbagrm.2011.09.002 21964328

[B8] Diao W.-P. Snyder J. C. Wang S.-B. Liu J.-B. Pan B.-G. Guo G.-J. (2016). Genome-wide Identification and Expression Analysis of WRKY Gene Family in *Capsicum Annuum* L. Front. Plant Sci. 7, 211. 10.3389/fpls.2016.00211 26941768 PMC4763034

[B9] Dou L. Zhang X. Pang C. Song M. Wei H. Fan S. (2014). Genome-wide Analysis of the WRKY Gene Family in Cotton. Mol. Genet. Genomics 289, 1103–1121. 10.1007/s00438-014-0872-y 24942461

[B10] Eulgem T. Rushton P. J. Robatzek S. Somssich I. E. (2000). The WRKY Superfamily of Plant Transcription Factors. Trends Plant Sci. 5 (5), 199–206. 10.1016/S1360-1385(00)01600-9 10785665

[B11] Fan C. Yao H. Qiu Z. Ma H. Zeng B. (2018). Genome-wide Analysis of *Eucalyptus Grandis WRKY* Genes Family and Their Expression Profiling in Response to Hormone and Abiotic Stress Treatment. Gene 678, 38–48. 10.1016/j.gene.2018.08.003 30077764

[B12] Goel R. Pandey A. Trivedi P. K. Asif M. H. (2016). Genome-wide Analysis of the Musa WRKY Gene Family: Evolution and Differential Expression during Development and Stress. Front. Plant Sci. 7, 299. 10.3389/fpls.2016.00299 27014321 PMC4789551

[B13] He G. H. Xu J. Y. Wang Y. X. Liu J. M. Li P. S. Chen M. (2016). Drought-responsive WRKY Transcription Factor Genes TaWRKY1 and TaWRKY33 from Wheat Confer Drought And/or Heat Resistance in *Arabidopsis* . BMC Plant Biol. 16, 116–16. 10.1186/s12870-016-0806-4 27215938 PMC4877946

[B14] He H. Dong Q. Shao Y. Jiang H. Zhu S. Cheng B. (2012). Genome-wide Survey and Characterization of the *WRKY* Gene Family in *Populus trichocarpa* . Plant Cel Rep 31 (7), 1199–1217. 10.1007/s00299-012-1241-0 22371255

[B15] Huang S. Gao Y. Liu J. Peng X. Niu X. Fei Z. (2012). Genome-wide Analysis of WRKY Transcription Factors in *Solanum lycopersicum* . Mol. Genet. Genomics 287 (6), 495–513. 10.1007/s00438-012-0696-6 22570076

[B16] Huang X. Li K. Xu X. Yao Z. Jin C. Zhang S. (2015). Genome-wide Analysis of WRKY Transcription Factors in white Pear (*Pyrus Bretschneideri*) Reveals Evolution and Patterns under Drought Stress. BMC Genomics 16, 1104. 10.1186/s12864-015-2233-6 26704366 PMC4691019

[B17] Ishiguro S. Nakamura K. (1994). Characterization of a cDNA Encoding a Novel DNA-Binding Protein, SPF1, that Recognizes SP8 Sequences in the 5′ Upstream Regions of Genes Coding for Sporamin and β-amylase from Sweet Potato. Mol. Gen. Genet. 244 (6), 563–571. 10.1007/BF00282746 7969025

[B18] Jiang Y. Duan Y. Yin J. Ye S. Zhu J. Zhang F. (2014). Genome-wide Identification and Characterization of the *Populus* WRKY Transcription Factor Family and Analysis of Their Expression in Response to Biotic and Abiotic Stresses. J. Exp. Bot. 65, 6629–6644. 10.1093/jxb/eru381 25249073 PMC4246191

[B19] Jiao Z. Sun J. Wang C. Dong Y. Xiao S. Gao X. (2018). Genome-wide Characterization, Evolutionary Analysis of *WRKY* Genes in Cucurbitaceae Species and Assessment of its Roles in Resisting to Powdery Mildew Disease. PLoS ONE 13 (12), e0199851. 10.1371/journal.pone.0199851 30589839 PMC6307730

[B20] Jue D. Sang X. Liu L. Shu B. Wang Y. Liu C. (2018). Identification of *WRKY* Gene Family from *Dimocarpus Longan* and its Expression Analysis during Flower Induction and Abiotic Stress Responses. Int. J. Mol. Sci. 19 (8), 2169. 10.3390/ijms19082169 PMC6121330 30044387

[B21] Kang G. Yan D. Chen X. Yang L. Zeng R. (2021). HbWRKY82 , a Novel IIc WRKY Transcription Factor from Hevea Brasiliensis Associated with Abiotic Stress Tolerance and Leaf Senescence in Arabidopsis. Physiol. Plantarum 171 (1), 151–160. 10.1111/ppl.13238 33034379

[B22] Kermode A. R. (2005). Role of Abscisic Acid in Seed Dormancy. J. Plant Growth Regul. 24, 319–344. 10.1007/s00344-005-0110-2

[B23] Kumar S. Stecher G. Li M. Knyaz C. Tamura K. (2018). MEGA X: Molecular Evolutionary Genetics Analysis across Computing Platforms. Mol. Biol. Evol. 35, 1547–1549. 10.1093/molbev/msy096 29722887 PMC5967553

[B25] Li J. Besseau S. Törönen P. Sipari N. Kollist H. Holm L. (2013). Defense‐related Transcription Factors WRKY 70 and WRKY 54 Modulate Osmotic Stress Tolerance by Regulating Stomatal Aperture in A Rabidopsis. New Phytol. 200 (2), 457–472. 10.1111/nph.12378 23815736 PMC4284015

[B26] Li X. Tang Y. Zhou C. Zhang L. Lv J. (2020). A Wheat WRKY Transcription Factor TaWRKY46 Enhances Tolerance to Osmotic Stress in Transgenic *Arabidopsis* Plants. Int. J. Mol. Sci. 21 (4), 1321. 10.3390/ijms21041321 PMC7072902 32075313

[B27] Ling J. Jiang W. Zhang Y. Yu H. Mao Z. Gu X. (2011). Genome-wide Analysis of WRKY Gene Family in Cucumis Sativus. BMC Genomics 12, 471. 10.1186/1471-2164-12-471 21955985 PMC3191544

[B28] Liu H. Sun M. Du D. Pan H. Cheng T. Wang J. (2015). Whole-transcriptome Analysis of Differentially Expressed Genes in the Vegetative Buds, floral Buds and Buds of chrysanthemum Morifolium. PLoS ONE 10, e0128009. 10.1371/journal.pone.0128009 26009891 PMC4444331

[B30] Luo X. Sun X. Liu B. Zhu D. Bai X. Cai H. (2013). Ectopic Expression of a WRKY Homolog from *Glycine Soja* Alters Flowering Time in *Arabidopsis* . PLoS ONE 8, e73295. 10.1371/journal.pone.0073295 23991184 PMC3753250

[B31] Meng D. Li Y. Bai Y. Li M. Cheng L. (2016). Genome-wide Identification and Characterization of WRKY Transcriptional Factor Family in Apple and Analysis of Their Responses to Waterlogging and Drought Stress. Plant Physiol. Biochem. 103, 71–83. 10.1016/j.plaphy.2016.02.006 26970718

[B32] Ning P. Liu C. Kang J. Lv J. (2017). Genome-wide Analysis of WRKY Transcription Factors in Wheat (*Triticum aestivum* L.) and Differential Expression under Water Deficit Condition. PeerJ 5, e3232. 10.7717/peerj.3232 28484671 PMC5420200

[B33] Phukan U. J. Jeena G. S. Shukla R. K. (2016). WRKY Transcription Factors: Molecular Regulation and Stress Responses in Plants. Front. Plant Sci. 7, 760. 10.3389/fpls.2016.00760 27375634 PMC4891567

[B34] Ramamoorthy R. Jiang S.-Y. Kumar N. Venkatesh P. N. Ramachandran S. (2008). A Comprehensive Transcriptional Profiling of the WRKY Gene Family in rice under Various Abiotic and Phytohormone Treatments. Plant Cel Physiol 49 (6), 865–879. 10.1093/pcp/pcn061 18413358

[B35] Ross C. A. Liu Y. Shen Q. J. (2007). The *WRKY* Gene Family in rice (*Oryza Sativa*). J. Integr. Plant Biol. 49 (6), 827–842. 10.1111/j.1672-9072.2007.00504.x

[B36] Rushton P. J. Somssich I. E. Ringler P. Shen Q. J. (2010). WRKY Transcription Factors. Trends Plant Sci. 15, 247–258. 10.1016/j.tplants.2010.02.006 20304701

[B37] Ryu H. Cho Y.-G. (2015). Plant Hormones in Salt Stress Tolerance. J. Plant Biol. 58, 147–155. 10.1007/s12374-015-0103-z

[B38] Singh A. K. Kumar S. R. Dwivedi V. Rai A. Pal S. Shasany A. K. (2017). A WRKY Transcription Factor from *Withania Somnifera* Regulates Triterpenoid Withanolide Accumulation and Biotic Stress Tolerance through Modulation of Phytosterol and Defense Pathways. New Phytol. 215 (3), 1115–1131. 10.1111/nph.14663 28649699

[B39] Singh K. Foley R. C. Oñate-Sánchez L. (2002). Transcription Factors in Plant Defense and Stress Responses. Curr. Opin. Plant Biol. 5, 430–436. 10.1016/S1369-5266(02)00289-3 12183182

[B40] Song H. Wang P. Lin J.-Y. Zhao C. Bi Y. Wang X. (2016). Genome-wide Identification and Characterization of *WRKY* Gene Family in Peanut. Front. Plant Sci. 7, 534. 10.3389/fpls.2016.00534 27200012 PMC4845656

[B41] Sun W. Ma Z. Chen H. Liu M. (2019). MYB Gene Family in Potato (Solanum tuberosum L.): Genome-wide Identification of Hormone-Responsive Reveals Their Potential Functions in Growth and Development. Int. J. Mol. Sci. 20, 4847. 10.3390/ijms20194847 PMC6801432 31569557

[B42] Thompson J. D. Higgins D. G. Gibson T. J. (1994). CLUSTAL W: Improving the Sensitivity of Progressive Multiple Sequence Alignment through Sequence Weighting, Position-specific gap Penalties and Weight Matrix Choice. Nucl. Acids Res. 22, 4673–4680. 10.1093/nar/22.22.4673 7984417 PMC308517

[B43] Ülker B. Somssich I. E. (2004). WRKY Transcription Factors: from DNA Binding towards Biological Function. Curr. Opin. Plant Biol. 7, 491–498. 10.1016/j.pbi.2004.07.012 15337090

[B44] Wan X. Zou L.-H. Zheng B.-Q. Tian Y.-Q. Wang Y. (2018). Transcriptomic Profiling for Prolonged Drought in Dendrobium Catenatum. Sci. Data 5, 180233. 10.1038/sdata.2018.233 30375990 PMC6207065

[B45] Wang C.-T. Ru J.-N. Liu Y.-W. Yang J.-F. Li M. Xu Z.-S. (2018). The maize WRKY Transcription Factor ZmWRKY40 Confers Drought Resistance in Transgenic *Arabidopsis* . Int. J. Mol. Sci. 19 (9), 2580. 10.3390/ijms19092580 PMC6164628 30200246

[B46] Wang D. Wang L. Su W. Ren Y. You C. Zhang C. (2020). A Class III WRKY Transcription Factor in Sugarcane Was Involved in Biotic and Abiotic Stress Responses. Sci. Rep. 10, 20964. 10.1038/s41598-020-78007-9 33262418 PMC7708483

[B47] Wang L. Liu F. Zhang X. Wang W. Sun T. Chen Y. (2018). Expression Characteristics and Functional Analysis of the *ScWRKY3* Gene from Sugarcane. Int. J. Mol. Sci. 19, 4059. 10.3390/ijms19124059 PMC6321069 30558233

[B48] Wang X. Li J. Guo J. Qiao Q. Guo X. Ma Y. (2020). The WRKY Transcription Factor PlWRKY65 Enhances the Resistance of *Paeonia Lactiflora* (Herbaceous Peony) to *Alternaria Tenuissima* . Hortic. Res. 7 (1), 57. 10.1038/s41438-020-0267-7 32284869 PMC7113260

[B49] Wei Y. Shi H. Xia Z. Tie W. Ding Z. Yan Y. (2016). Genome-wide Identification and Expression Analysis of the *WRKY* Gene Family in Cassava. Front. Plant Sci. 7, 25. 10.3389/fpls.2016.00025 26904033 PMC4742560

[B50] Wu Z.-J. Li X.-H. Liu Z.-W. Li H. Wang Y.-X. Zhuang J. (2016). Transcriptome-wide Identification of *Camellia Sinensis* WRKY Transcription Factors in Response to Temperature Stress. Mol. Genet. Genomics 291 (1), 255–269. 10.1007/s00438-015-1107-6 26308611

[B51] Xie Z. Zhang Z.-L. Zou X. Huang J. Ruas P. Thompson D. (2005). Annotations and Functional Analyses of the Rice WRKY Gene Superfamily Reveal Positive and Negative Regulators of Abscisic Acid Signaling in Aleurone Cells. Plant Physiol. 137, 176–189. 10.1104/pp.104.054312 15618416 PMC548849

[B52] Yamaguchi-Shinozaki K. Shinozaki K. (2005). Organization of *Cis*-Acting Regulatory Elements in Osmotic- and Cold-Stress-Responsive Promoters. Trends Plant Sci. 10 (2), 88–94. 10.1016/j.tplants.2004.12.012 15708346

[B53] Yang B. Jiang Y. Rahman M. H. Deyholos M. K. Kav N. N. (2009). Identification and Expression Analysis of WRKY Transcription Factor Genes in Canola (*Brassica Napus* L.) in Response to Fungal Pathogens and Hormone Treatments. BMC Plant Biol. 9, 68. 10.1186/1471-2229-9-68 19493335 PMC2698848

[B54] Yang X. Li H. Yang Y. Wang Y. Mo Y. Zhang R. (2018). Identification and Expression Analyses of *WRKY* Genes Reveal Their Involvement in Growth and Abiotic Stress Response in Watermelon (*Citrullus lanatus*). PLoS ONE 13 (1), e0191308. 10.1371/journal.pone.0191308 29338040 PMC5770075

[B55] Zhang G.-Q. Xu Q. Bian C. Tsai W.-C. Yeh C.-M. Liu K.-W. (2016). The *Dendrobium Catenatum* Lindl. Genome Sequence Provides Insights into Polysaccharide Synthase, floral Development and Adaptive Evolution. Sci. Rep. 6, 19029. 10.1038/srep19029 26754549 PMC4709516

[B56] Zhang T. Cui Z. Li Y. Kang Y. Song X. Wang J. (2021). Genome-wide Identification and Expression Analysis of MYB Transcription Factor Superfamily in *Dendrobium Catenatum* . Front. Genet. 12, 714696. 10.3389/fgene.2021.714696 34512725 PMC8427673

[B57] Zhang Y. Wang L. (2005). The WRKY Transcription Factor Superfamily: its Origin in Eukaryotes and Expansion in Plants. BMC Evol. Biol. 5, 1. 10.1186/1471-2148-5-1 15629062 PMC544883

[B59] Zhou C. Lin Q. Lan J. Zhang T. Liu X. Miao R. (2020). WRKY Transcription Factor OsWRKY29 Represses Seed Dormancy in rice by Weakening Abscisic Acid Response. Front. Plant Sci. 11, 691. 10.3389/fpls.2020.00691 32536934 PMC7268104

[B60] Zhu H. Jiang Y. Guo Y. Huang J. Zhou M. Tang Y. (2021). A Novel Salt Inducible WRKY Transcription Factor Gene, *AhWRKY75*, Confers Salt Tolerance in Transgenic Peanut. Plant Physiol. Biochem. 160, 175–183. 10.1016/j.plaphy.2021.01.014 33497848

[B61] Zhu J.-K. (2016). Abiotic Stress Signaling and Responses in Plants. Cell 167 (2), 313–324. 10.1016/j.cell.2016.08.029 27716505 PMC5104190

[B62] Zotz G. Winkler U. (2013). Aerial Roots of Epiphytic Orchids: The Velamen Radicum and its Role in Water and Nutrient Uptake. Oecologia 171, 733–741. 10.1007/s00442-012-2575-6 23292456

