# Supplementary figures and images for: Identification and Expression Analysis of WRKY Gene Family in Response to Abiotic Stress in Dendrobium catenatum
Source: Front Genet. 2022 Feb 3;13:800019. doi: 10.3389/fgene.2022.800019 (PMC8850645; doi:10.3389/fgene.2022.800019)

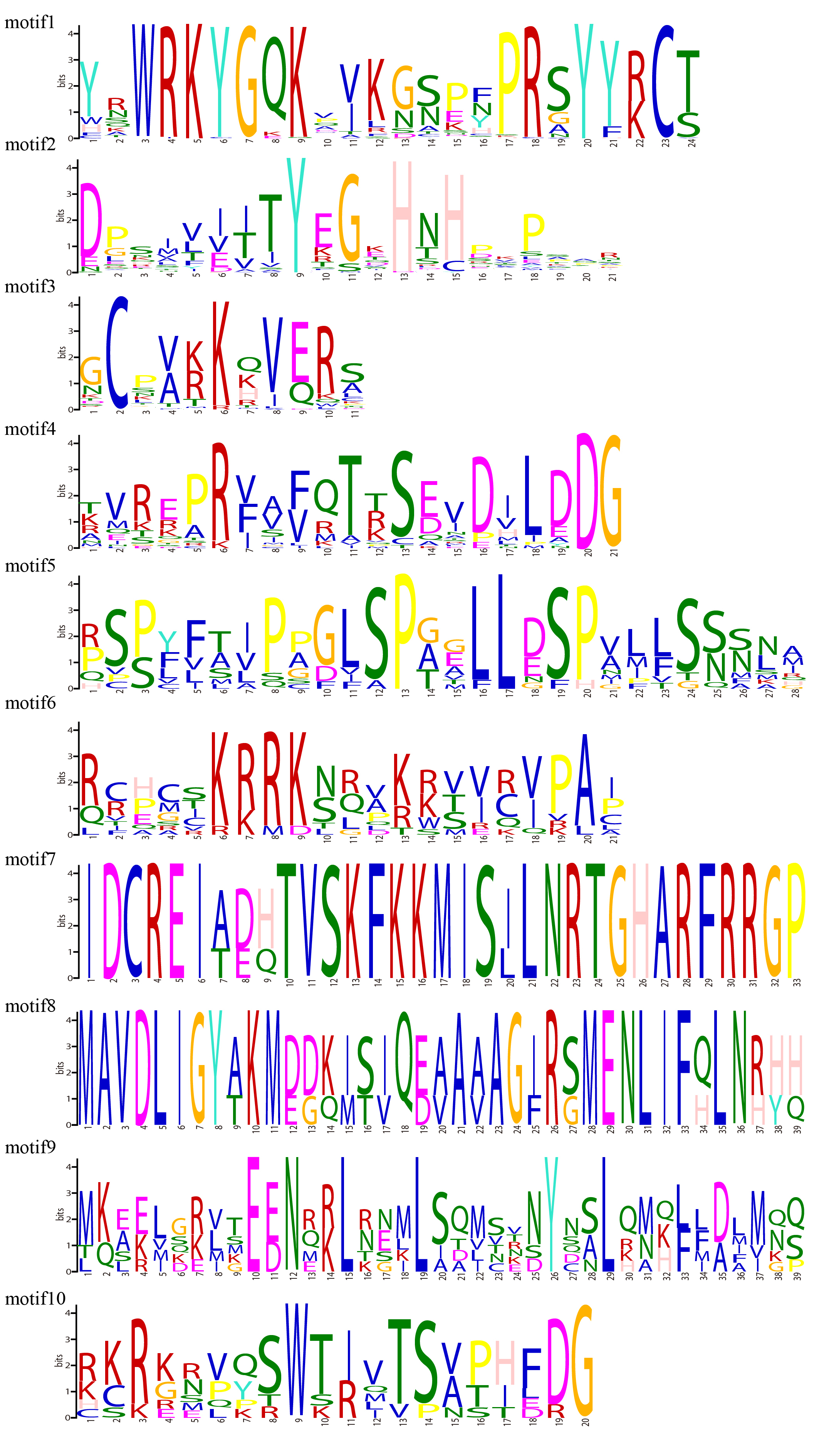

Supplement: Supplementary file 2 [file Image3.JPEG]

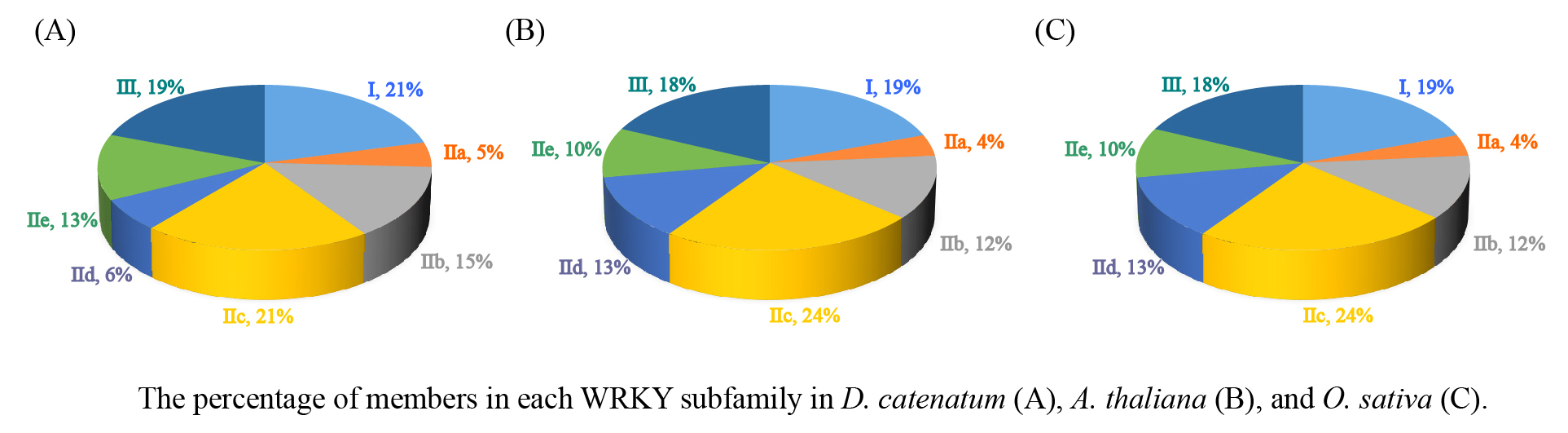

Supplement: Supplementary file 5 [file Image1.JPEG]

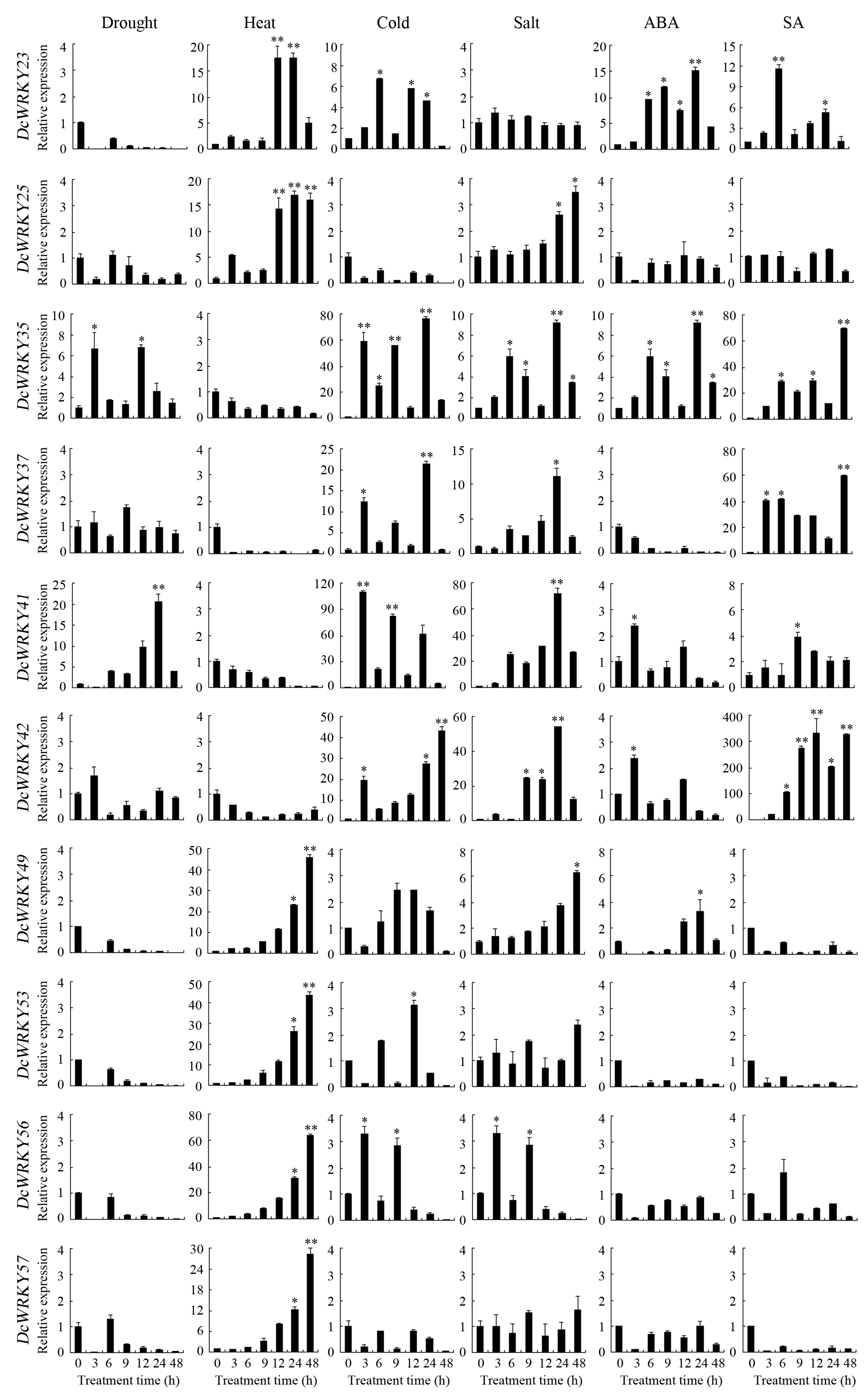

Supplement: Supplementary file 6 [file Image4.JPEG]

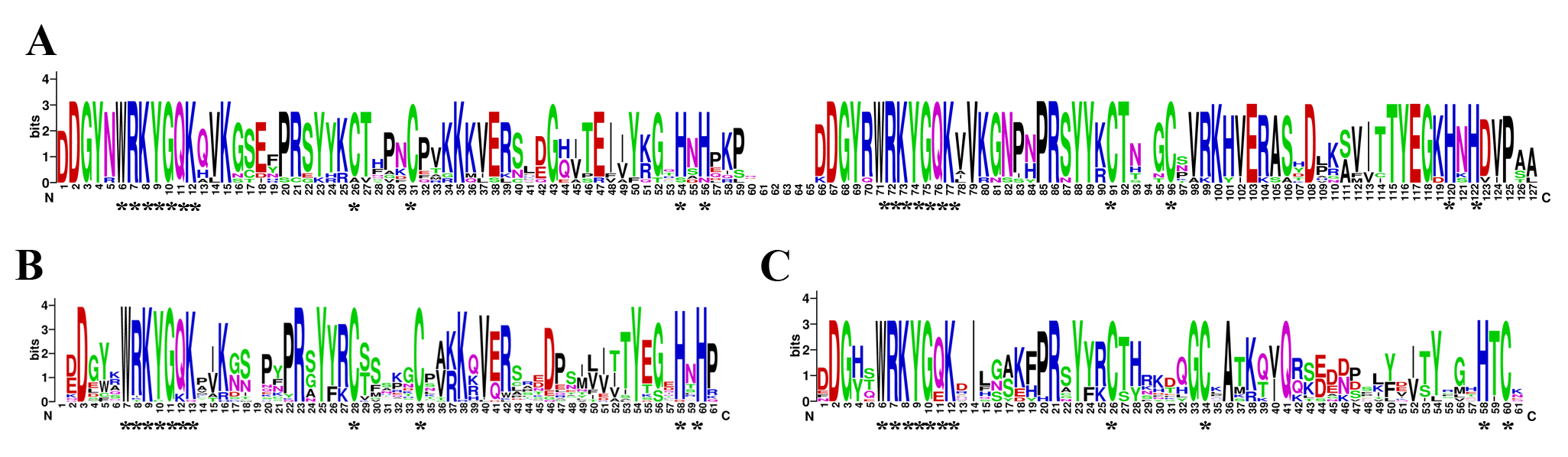

Supplement: Supplementary file 8 [file Image2.JPEG]

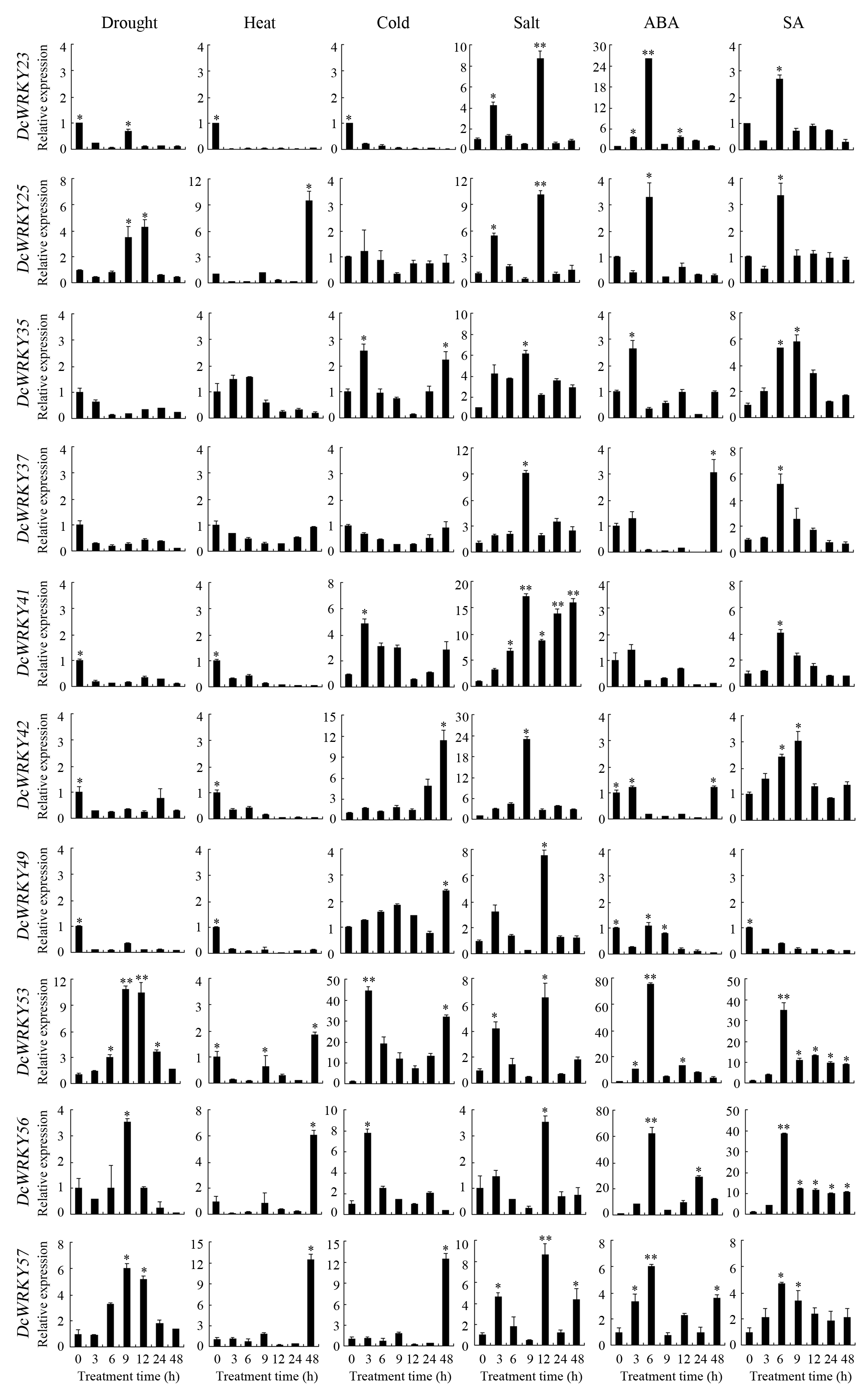

Supplement: Supplementary file 9 [file Image5.JPEG]
