# Supplementary material for: Identification and Expression Analysis of WRKY Gene Family in Response to Abiotic Stress in Dendrobium catenatum
Source: Front Genet. 2022 Feb 3;13:800019. doi: 10.3389/fgene.2022.800019 (PMC8850645; doi:10.3389/fgene.2022.800019)
Supplement: Supplementary file 14 [file DataSheet1.docx]

Supplementary Material

**Supplementary Figure S1** The percentage of members in each WRKY subfamily in *D. catenatum* (A), *A. thaliana* (B), and *O. sativa* (C).

**Supplementary Figure S2** The WRKY domain sequences of different groups in *D. catenatum*. The logos of the group I **(A)**, group II **(B),** and group III **(C)** are based on the sequence alignments of *D. catenatum* WRKY domains. The bit score indicates the information content for each position in the sequence. Stars indicate the conserved WRKYGQK sequence and the zinc-finger motif (C_2_H_2_ or C_2_HC).

**Supplementary Figure S3** The amino acid sequences of each motif identified in DcWRKY proteins.

**Supplementary Figure S4** Real-time quantitative PCR analysis of 10 *DcWRKY* genes under abiotic stress and hormone treatment in roots. Vertical bars indicate the standard deviation (n=3). Values of 0, 3, 6, 9, 12, 24, and 48 indicate hours after treatment. The unstressed level (0 h) was used as a control. Asterisk (∗ or ∗∗) indicate a significant difference at P <0.05 or 0.01, respectively.

**Supplementary Figure S5** Real-time quantitative PCR analysis of 10 *DcWRKY* genes under abiotic stress and hormone treatment in leaves. Vertical bars indicate the standard deviation (n=3). Values of 0, 3, 6, 9, 12, 24, and 48 indicate hours after treatment. The unstressed level (0 h) was used as a control. Asterisk (∗ or ∗∗) indicate a significant difference at P <0.05 or 0.01, respectively.

**Supplementary Table S1** The gene ID of *WRKY* genes in *D. catenatum*, *A. thaliana* and *O. sativa*.

**Supplementary Table S2** Primers used in this study.

**Supplementary Table S3** Physicochemical properties of *D. catenatum* WRKY proteins.

**Supplementary** **Table S4** The genomic, CDS and protein sequences of *DcWRKY* genes

**Supplementary Table S5** Motifs and the number of introns of each *DcWRKY* gene.

**Supplementary Table S6** Information on *cis*-elements of *DcWRKY* gene promoters.

**Supplementary Table S7** Expression of *DcWRKY* genes under drought stress (TPM).

**Supplementary Table S8** Expression of selected *DcWRKY* genes in different tissues by RT-qPCR.
